# Association of Greenness with Blood Pressure among Individuals with Type 2 Diabetes across Rural to Urban Community Types in Pennsylvania, USA

**DOI:** 10.3390/ijerph18020614

**Published:** 2021-01-13

**Authors:** Melissa N. Poulsen, Brian S. Schwartz, Cara Nordberg, Joseph DeWalle, Jonathan Pollak, Giuseppina Imperatore, Carla I. Mercado, Karen R. Siegel, Annemarie G. Hirsch

**Affiliations:** 1 Department of Population Health Sciences, Geisinger, Danville, PA 17822, USA; bschwar1@jhu.edu (B.S.S.); cmnordberg@geisinger.edu (C.N.); jjdewalle@geisinger.edu (J.D.); aghirsch@geisinger.edu (A.G.H.); 2 Department of Environmental Health and Engineering, Johns Hopkins Bloomberg School of Public Health, Baltimore, MD 21205, USA; jpollak2@jhu.edu; 3 Department of Epidemiology, Johns Hopkins Bloomberg School of Public Health, Baltimore, MD 21205, USA; 4 Department of Medicine, Johns Hopkins School of Medicine, Baltimore, MD 21205, USA; 5 Division of Diabetes Translation, National Center for Chronic Disease Prevention and Health Promotion, Centers for Disease Control and Prevention, Atlanta, GA 30333, USA; gai5@cdc.gov (G.I.); wif5@cdc.gov (C.I.M.); yuo0@cdc.gov (K.R.S.)

**Keywords:** community context, diabetes mellitus, greenspace, hypertension, percent forest, rural health

## Abstract

Greenness may impact blood pressure (BP), though evidence is limited among individuals with type 2 diabetes (T2D), for whom BP management is critical. We evaluated associations of residential greenness with BP among individuals with T2D in geographically diverse communities in Pennsylvania. To address variation in greenness type, we evaluated modification of associations by percent forest. We obtained systolic (SBP) and diastolic (DBP) BP measurements from medical records of 9593 individuals following diabetes diagnosis. Proximate greenness was estimated within 1250-m buffers surrounding individuals’ residences using the normalized difference vegetation index (NDVI) prior to blood pressure measurement. Percent forest was calculated using the U.S. National Land Cover Database. Linear mixed models with robust standard errors accounted for spatial clustering; models were stratified by community type (townships/boroughs/cities). In townships, the greenest communities, an interquartile range increase in NDVI was associated with reductions in SBP of 0.87 mmHg (95% CI: −1.43, −0.30) and in DBP of 0.41 mmHg (95% CI: −0.78, −0.05). No significant associations were observed in boroughs or cities. Evidence for modification by percent forest was weak. Findings suggest a threshold effect whereby high greenness may be necessary to influence BP in this population and support a slight beneficial impact of greenness on cardiovascular disease risk.

## 1. Introduction

The natural environment—including greenness—is hypothesized to benefit health through multiple, synergistic pathways [1], which are organized into three domains [2]. The *restorative capacity* of natural environments is thought to reduce stress, as originally theorized by the Psychoevolutionary Stress Reduction Theory, which posits that nature affects emotional and physiological outcomes through stress-reducing qualities [3]. Natural environments also *build capacity* for health and well-being by encouraging health-promoting behaviors through provision of restorative spaces conducive to physical activity and social interaction, and they *reduce harm* through reduced exposure to environmental stressors, including air pollution, heat, and noise [2]. An extensive body of research has examined greenness in relation to health and health behaviors, but evidence for an effect of greenness on cardiovascular health remains limited [4]. Identifying community characteristics, such as greenness, that influence cardiovascular health can serve as a resource for clinical decision-making as well as inform population-level intervention efforts [5].

To date, most epidemiologic research on greenness and cardiovascular health has been conducted among general population samples [6]. As such, a question remains as to whether the natural environment confers the same beneficial effects when cardiovascular health is already at risk. Experimental studies of *shinrin-yoku* (“forest bathing,” or taking in the forest through the senses) that have been conducted among individuals with chronic diseases such as hypertension, chronic obstructive pulmonary disease, and congestive heart failure have demonstrated therapeutic effects [7]. To our knowledge, however, no studies have evaluated greenness and measures of cardiovascular health, such as blood pressure, among individuals with type 2 diabetes (T2D). Blood pressure management is critical for this group. Among individuals with T2D, high blood pressure amplifies the risk for cardiovascular disease and microvascular complications such as chronic kidney disease and retinopathy, whereas management of blood pressure among individuals with diabetes reduces such risks [8,9,10], representing an important target for prevention. Such management involves clinical treatment as well as behavioral modifications that may be influenced by community characteristics [11], including the natural environment. 

One critique of past literature on greenness and health is the lack of consideration of variation in greenness type (e.g., forest, agricultural land), which has contributed to a mixed and inconclusive evidence base regarding the impact of greenness on health [12]. (Multiple, often poorly-defined terms are used to denote areas of vegetation in a landscape [13]; here we use “greenness” as a broad term to reference such areas, which can include “greenspaces” such as parks.) Different types of greenness have varying capacities to influence health, dependent on the mechanism connecting greenness with a particular health outcome [1,12]. Forests, which are associated with greater feelings of restoration than other natural environments [14] and release potentially health-benefitting phytoncides [15,16], may have more salutogenic effects than other vegetated areas such as agricultural land [17]. 

As part of an epidemiological study on determinants of geographic disparities in T2D, we evaluated associations of greenness with blood pressure among a cohort of individuals with T2D. Considering the varying degrees of greenness by community type (and concomitant opportunities for greenness exposure), we evaluated a geographically diverse range of communities in Pennsylvania that spanned the urban to rural continuum. The first analysis goal was to evaluate the relationship between residential greenness and blood pressure early in the course of T2D, when community characteristics may potentially have more influence on the disease course. To address the lack of consideration of variation in greenness type, the second analysis goal was to evaluate effect modification by percent of forested land surrounding the residence on associations of greenness with blood pressure. 

## 2. Materials and Methods 

This retrospective cohort study was conducted by Geisinger-Johns Hopkins Bloomberg School of Public Health, one of four academic research centers in the Diabetes LEAD (Location, Environmental Attributes, and Disparities) Network (http://diabetesleadnetwork.org/), a collaboration funded by the Centers for Disease Control and Prevention dedicated to providing scientific evidence to develop targeted community-based interventions and policies to prevent incident T2D and related health outcomes across the United States [18].

### 2.1. Study Population

**Figure 1:**
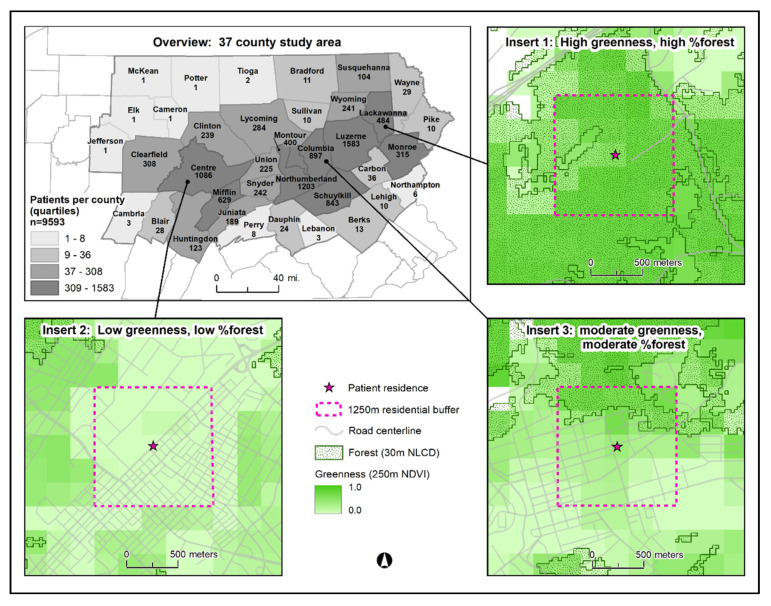
Map of 37-county study area in Pennsylvania, USA. Numbers within each county indicate the number of study individuals per county. Inserts provide three examples depicting varying degrees of greenness and percent forest in buffers surrounding patient residences. Abbreviations: NLCD, National Land Cover Database; NDVI, Normalized Difference Vegetation Index.

We obtained electronic health record data for 15,888 Geisinger patients diagnosed with T2D between 2008–2016 who resided in the 37-county study area (Figure 1). Geisinger, an integrated health system, serves central and northeast Pennsylvania and has a primary care population representative of the age and sex distribution of the region’s general population [19]. As previously described [20], among individuals with at least two visits to a Geisinger primary care provider, we identified individuals as having T2D if they had at least two encounters with a T2D diagnosis based on Epic (Verona, WI) electronic diagnosis group names (more granular clinical terms selected by clinicians for diagnosis during encounters) or International Classification of Diseases [ICD] 9th and 10th Revision codes (ICD-9: 250.00, 250.02, 250.10, 250.12, 250.20, 250.22, 250.30, 250.32, 250.40, 250.42, 250.50, 250.52, 250.60, 250.62, 250.70, 250.72, 250.80, 250.82, 250.90, 250.92; ICD-10: E11.xx), at least one T2D medication order (other than metformin or acarbose if female), or at least one encounter with a T2D diagnosis and an abnormal laboratory value for a glucose or hemoglobin A1c test (random glucose ≥ 200 mg/dL; fasting glucose ≥ 126 mg/dL, or hemoglobin A1c ≥ 6.5%). Individuals with a diagnosis for type 1 diabetes and women with gestational diabetes were excluded. To exclude prevalent cases of T2D, we only included individuals who had electronic health record data at least two years prior to diabetes diagnosis and who did not meet the T2D criteria during this two-year observation period. In this analysis, “diagnosis” refers to the date that cases first met the T2D criteria. 

We excluded children (<18 years of age) and individuals with conditions that severely impact blood pressure, including a diagnosis with secondary hypertension (hypertension due to another condition such as kidney disease) or indication of severe renal disease (i.e., kidney transplant, end-stage renal disease, receiving dialysis, estimated glomerular filtration rate <30 mL/min).

### 2.2. Electronic Health Record Measures

#### 2.2.1. Blood Pressure

Study outcomes included systolic blood pressure (SBP) and diastolic blood pressure (DBP), taken from the same clinical blood pressure measurement. To evaluate blood pressure early in the course of T2D, we selected one blood pressure measurement per individual from an outpatient primary care visit during the second year after meeting the criteria for T2D diagnosis. Blood pressure measurements were obtained during the second year following T2D diagnosis, rather than the year of diagnosis, to allow time for diabetes and antihypertensive treatment to stabilize. In a sensitivity analysis, we analyzed blood pressure measurements during the third year following T2D diagnosis.

Blood pressure measurements were assumed to be taken per usual care. Workflow guidelines dictate patients should be sitting in a chair with their feet on the floor for at least five minutes prior to blood pressure measurement; the cuff should be placed at heart level, with the lower edge of the cuff 2–3 cm above antecubital space, on an arm free of clothing and using an appropriately-sized cuff. If an individual had more than one outpatient visit with a blood pressure measurement during the one-year period, we randomly selected one measurement. If there were two blood pressure measurements taken on the same day within 15 min of one another, we used the second measurement, as clinical protocol requires a second blood pressure measurement after an initial elevated value. Blood pressure measurements were not selected within 30 days of a hospitalization, nor from women during pregnancy through six weeks post-partum. We did not use extreme values that could reflect acute, severe illness or non-sensical values (SBP < 60 or >250 mmHg; DBP < 40 or >140 mmHg).

Of the 15,888 individuals with T2D diagnosis between 2008 and 2016, we obtained blood pressure measurements for 10,383 individuals in the second year following T2D diagnosis; the remaining 5505 did not have a qualifying outpatient blood pressure measurement (e.g., T2D diagnosis occurred in 2015 or 2016) and were therefore excluded from analysis. For the sensitivity analysis, 7603 individuals had a qualifying blood pressure measurement in the third year following T2D diagnosis.

#### 2.2.2. Individual Covariates

From electronic health records, we obtained hypothesized confounding variables, including current blood pressure medication use (i.e., antihypertensives including ACE inhibitors, angiotensin II receptor antagonists, beta blockers, calcium channel blockers, and diuretics), primary hypertension diagnosis (prior to, or within 30 days following the date of the blood pressure measurement), body mass index (BMI, kg/m^2^), smoking status, age, sex, race, ethnicity, use of Medical Assistance (Pennsylvania’s needs-based insurance, which serves as a proxy for low family socioeconomic status [21]), and season of the blood pressure measurement. Regarding blood pressure medications, a critical covariate in a study of blood pressure, we observed inconsistencies in how medications were documented in different fields of structured data in the electronic health record, which led us to develop a more rigorous process to more accurately identify study individuals’ current blood pressure medication use (Figure S1).

### 2.3. Environmental Measures

We used ESRI ArcGIS 10.4 (ESRI Inc., Redlands, CA, USA) to geocode study individuals, assign the residential address to a community, create environmental measures, and link individuals’ residential addresses to environmental measures. Communities were defined using previously-evaluated boundaries [22] that we have employed in prior research in the study region (e.g., [20,23]). The approach combines Pennsylvania’s minor civil divisions—which represent behaviorally- and policy-relevant boundaries—with city census tracts, thereby providing relevant spatial resolutions to divide densely populated cities. These administrative community types included townships (rural/suburban areas), boroughs (small towns), and city census tracts (urban) and represent a continuum of lower to higher population density and land use mix [22].

#### 2.3.1. Proximate Greenness

Similar to Yeager and colleagues [24], who evaluated contemporaneous greenness in relation to cardiovascular disease biomarkers, we evaluated proximate greenness by estimating normalized difference vegetation index (NDVI) immediately prior to the date of the blood pressure measurement. We used proximate greenness rather than peak or cumulative greenness because we expected an acute temporal relationship between greenness—which differs between winter and summer months in Pennsylvania—and blood pressure. This metric also addressed temporality requirements. NDVI quantifies vegetation reflectance in 16-day composite periods based on satellite images from the Moderate-resolution Imaging Spectroradiometer (MODIS) from NASA’s Aqua satellite [25]. The index ranges from −1.0 to 1.0 (higher values indicating higher greenness) and aggregates all types of greenness (forest, lawns, agricultural land, wetlands, etc.). We calculated average greenness in the composite period immediately preceding the blood pressure measurement date in 1250-m by 1250-m square buffers surrounding study individuals’ home addresses (Figure 1 inserts). If this composite was missing (e.g., due to cloudiness), we used the average NDVI value in the 16-day composite immediately preceding the missing composite (6% of observations). We excluded individuals from the study for whom both composites were missing (n = 189, 2% of observations for the primary analysis; n = 286, 4% of observations for the sensitivity analysis of blood pressure measurements during the third year following T2D diagnosis). 

#### 2.3.2. Percent Forest

We estimated study individuals’ exposure to forested areas using the most recent release of data (2006, 2011) from the U.S. National Land Cover Database (NLCD) prior to the date of their blood pressure measurement. Percent forested area was calculated using NLCD land classes for deciduous, evergreen, and mixed forest within each buffer (Figure 1 inserts). To create geographical boundaries matching the native greenness pixel resolution, a circular buffer with a radius of 463-m was created around each individual’s residence, then converted to the 1250-m by 1250-m square buffers using the “Feature Envelope to Polygon” tool in ArcMap. 

#### 2.3.3. Community Socioeconomic Deprivation

Community socioeconomic deprivation (CSD) was evaluated as a potential confounder of greenness and blood pressure associations. CSD was derived from summed z-scores of six sociodemographic indicators from the American Community Survey (2006–2011, 2011–2015) [26]: proportions of the population with less than high school education, unemployed, not in labor force, in poverty, receiving public assistance, and households without a car [27].

### 2.4. Statistical Analyses

The goals of the analysis were twofold. First, to evaluate associations (reported as beta coefficients with 95% confidence intervals [CI]) of proximate greenness and blood pressure during the second year following T2D diagnosis, we used linear mixed models with robust standard errors to account for clustering of individuals within communities. We assessed potential nonlinearity in greenness associations by evaluating linear, quadratic, and cubic terms for NDVI. Similar to a prior study [28], to enhance model interpretability, effect estimates of greenness are reported by the difference in the interquartile range; the resulting coefficients represented the differences in blood pressure comparing individuals at the 75th percentile (i.e., average “high”) versus the 25th percentile (average “low”) of greenness. Due to non-overlapping distributions of environmental measures across community types, models were stratified by administrative community type to avoid positivity violations that occur if data are pooled from heterogenous places [29]. Models were adjusted for variables identified a priori as likely confounders, including age (continuous, in years, centered), sex, race (white versus all other racial groups), ethnicity (Hispanic versus non-Hispanic), use of Medical Assistance (ever versus never), smoking status (current, former, never, unknown), current blood pressure medication usage (yes versus no), and primary hypertension status (yes versus no). We assessed nonlinearity of the association between age and blood pressure by evaluating linear, quadratic, and cubic age terms. Final SBP models adjusted for linear and quadratic age terms; DBP models further adjusted for the cubic term. We evaluated CSD (quartiled) as a potential confounder of greenness and blood pressure associations by adding it to models; these models excluded race and ethnicity variables due to issues with non-positivity. Multicollinearity was evaluated by examining variance inflation factors; influence, leverage, heteroscedasticity, and linearity were checked with added variable plots. To evaluate whether duration with T2D may impact associations of greenness and blood pressure, we conducted a sensitivity analysis that repeated the adjusted SBP and DBP models using blood pressure measurements taken during the third year following T2D diagnosis among the 7317 individuals with a qualifying third year blood pressure measurement and who were not missing NDVI values. We tested effect modification of greenness and year two blood pressure associations separately by two definitions of hypertension (primary hypertension diagnosis; current blood pressure medication usage), age categories (<40, 40–64, ≥65), and BMI categories (<30 versus ≥30; 189 individuals excluded due to missing BMI data). Effect modification was considered present if the interaction terms between greenness and the modifier were significant (*p* < 0.05). 

The second analysis goal was to evaluate whether associations of proximate greenness and blood pressure differed by percent forest. To do this, we added cross-product terms of proximate greenness (continuous) and percent forest (quartiled) to the linear regression models and assessed cross-product terms for statistical significance as well as their average marginal effects. Models adjusted for the same covariates described above and were stratified by administrative community type. 

Finally, because blood pressure is known to differ by season—with higher blood pressure occurring in winter months [30]—and season affects proximate greenness, we evaluated our data to try to disentangle potential confounding of proximate greenness and blood pressure associations by the season of blood pressure measurements. We evaluated distributions of proximate greenness by season. We also examined associations between season of blood pressure measurement and blood pressure stratified by community type, adjusting for the same set of covariates as in the greenness and blood pressure models. We could not analyze proximate greenness and season in the same model due to multicollinearity; instead, we evaluated greenness and blood pressure outcomes stratified by season of the blood pressure measurement and administrative community type. To simplify stratified analyses, season was dichotomized as winter versus non-winter; initial exploratory evaluation of specifications with more categories (e.g., four seasons) showed that primary differences in blood pressure occurred between winter and all other seasons. 

Analyses were conducted using Stata version 15.1 (StataCorp LP, College Station, TX, USA).

## 3. Results

### 3.1. Characteristics of Study Individuals and Communities 

From the 10,383 individuals with T2D with a qualifying blood pressure measurement during the second year following T2D diagnosis, 9593 met inclusion criteria (Table S1). The majority (97%) were white and non-Hispanic, reflecting the racial and ethnic composition of the study region (Table 1). A majority of the study population lived in townships (versus boroughs and city census tracts). Township study residents had lower participation in Medical Assistance and a lower proportion of current smokers. City study residents were more likely to be female, were slightly younger, and were more likely to have normal blood pressure and less likely to be on blood pressure medications or have a diagnosis of primary hypertension. 

**Figure 2:**
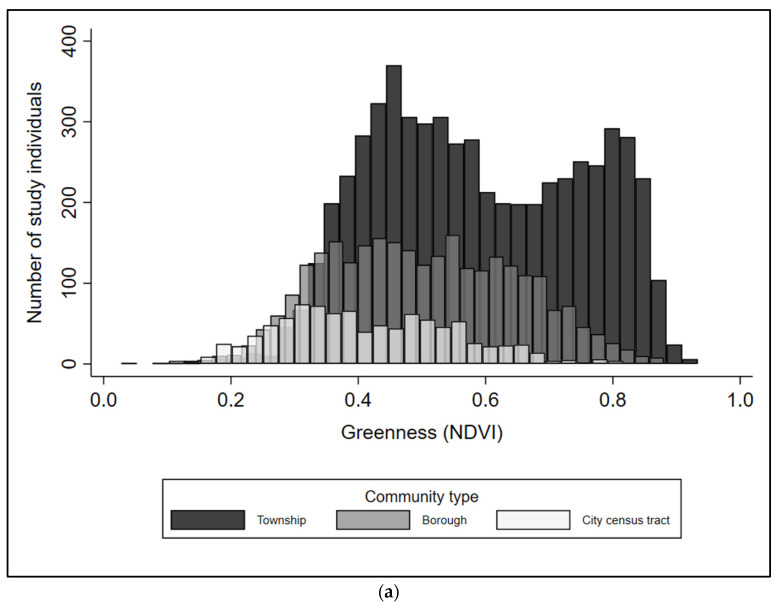

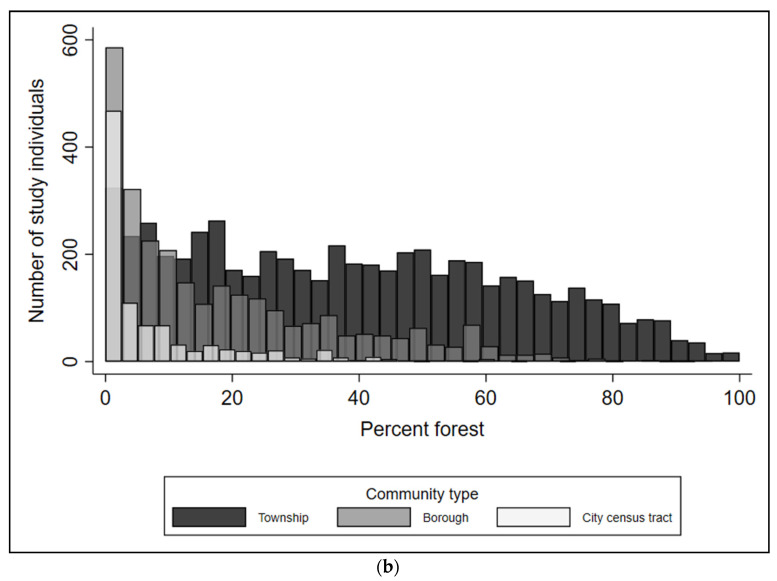
Distributions of study individuals by measures of (**a**) proximate greenness and (**b**) percent forest, overlaid across the three administrative community types. Abbreviations: NDVI, normalized difference vegetation index.

Proximate greenness and percent forest were highest and had the largest range within townships compared with boroughs and city census tracts (Figure 2). Greenness and percent forest were lowest in cities, and 21% of city residents and 5% of borough residents resided within a buffer with no forest.

### 3.2. Associations of Greenness and Blood Pressure 

In both unadjusted models and models adjusted for covariates, we observed that higher greenness was significantly associated with lower SBP and DBP during the second year following T2D diagnosis only in townships (Table 2; unadjusted results not shown). After adjustment, an interquartile range increase in NDVI was significantly associated with reductions in SBP of 0.87 mmHg (95% CI: −1.43, −0.30) and in DBP of 0.41 mmHg (95% CI: −0.78, −0.05) among township residents. The approximate effect size and direction of the association were similar among borough residents, but were not statistically significant. We observed no evidence of an association of greenness with blood pressure among city residents. 

Associations were consistent in a sensitivity analysis of the 7317 individuals with blood pressure measurements from the third year following T2D diagnosis. After adjustment for covariates, an interquartile range increase in NDVI was significantly associated with reductions in SBP of 1.05 mmHg (95% CI: −1.66, −0.43) and in DBP of 0.54 mmHg (−0.93, −0.16) among township residents. We observed no associations among borough or city residents (results not shown).

We observed no evidence of confounding by CSD, nor evidence of effect modification of greenness and year two blood pressure associations by primary hypertension status, current blood pressure medications, age categories, or BMI categories (results not shown). 

### 3.3. Effect Modification by Percent Forest on Associations of Greenness with Blood Pressure 

None of the greenness by percent forest cross-product terms were statistically significant in SBP or DBP models. However, evaluation of the average marginal effects from township models revealed a negative relationship between greenness and blood pressure in the fourth quartile of percent forest (SBP: −1.69 [−2.62, −0.76]; DBP: −0.62 [−1.18, −0.06]), but not in quartiles 1–3.

### 3.4. Disentangling Potential Confounding of Greenness and Blood Pressure Associations by Season

**Figure 3:**
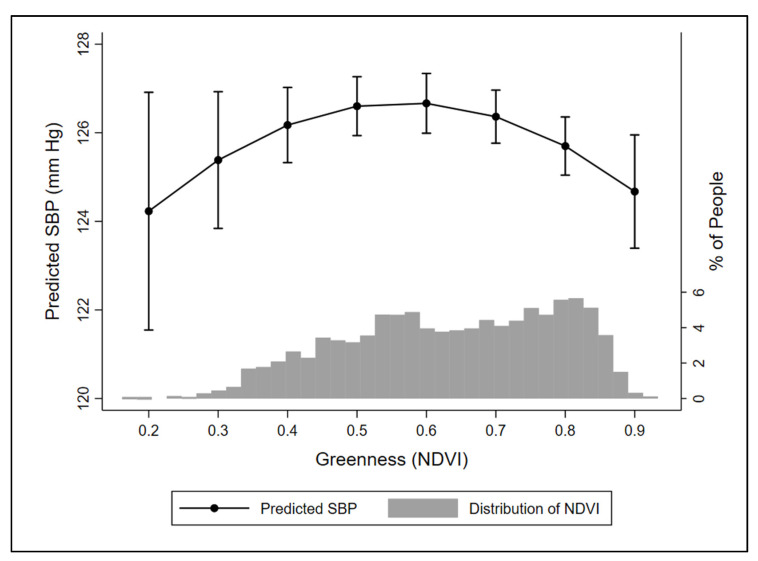
Predicted SBP (mmHg) with 95% confidence intervals by proximate greenness in townships in non-winter months (March–November). Histogram depicts the distribution of NDVI across study individuals. Abbreviations: NDVI, normalized difference vegetation index; SBP, systolic blood pressure.

Figure S2 shows the differing distributions of proximate greenness by season and community type. Compared with measurements in non-winter months, SBP in winter months was significantly higher on average in townships (1.40 [0.63, 2.17]) and boroughs (1.27 [0.21, 2.34]), but not cities (1.14 [−0.93, 3.21]). DBP measurements in winter months were significantly higher on average in townships (0.76 [0.27, 1.25]) and cities (1.61 [0.09, 3.12], but not boroughs (0.45 [−0.29, 1.20]). In models stratified by season (winter versus non-winter) we observed a nonlinear association of greenness and SBP in townships in non-winter (Figure 3), but no association in winter, for other community types, or for greenness with DBP.

## 4. Discussion

In this study, we evaluated associations of greenness and blood pressure in a cohort of individuals with T2D representing geographically diverse communities in Pennsylvania, accounting for important clinical confounders (hypertension and blood pressure medication use) and socioeconomic confounders (Medical Assistance, community socioeconomic deprivation). Higher proximate greenness was associated with slightly lower SBP and DBP in the second and third year following T2D diagnosis among residents of townships, the most rural administrative community type. Given the many factors influencing blood pressure, potential effects of the natural environment are likely to be obscured by individuals’ health behaviors and medical care, particularly among a population with high risk of having already established arterial disease. Although study individuals were early in the course of T2D, the majority had primary hypertension. Thus, our finding of even a small effect of greenness on blood pressure in townships is notable and adds to the evidence for a beneficial impact of greenness on cardiovascular disease risk.

A meta-analysis of greenness and health outcomes reported an association with blood pressure, with “high” (versus “low”) exposure to greenness related to lower DBP (−1.97 mmHg [95% CI: −3.45, −0.19]) and similar but non-significant inferences for SBP (−1.50 mmHg [−3.43, 0.44)] [6]. In a large cross-sectional study among urban Chinese adults, Yang and colleagues [28] reported a 0.82 mmHg reduction in SBP per interquartile range increase in greenness. Conducted among general population samples, these studies are not directly comparable to our findings; however, our findings showed comparable effect sizes. In townships, individuals in the 75th percentile of greenness had 0.87 mmHg lower SBP and 0.41 mmHg lower DBP compared with those in the 25th percentile. Though such small differences in blood pressure are not likely to be clinically meaningful, they can have important public health impacts. More than 25% of individuals with diabetes do not meet blood pressure goals, despite the estimated 87% who use antihypertensive medications [31]. A modest population-wide reduction in SBP of 1–2 mmHg could prevent substantial numbers of adverse cardiovascular events, highlighting the importance of complementing clinical blood pressure management strategies with population-level interventions [32].

Associations of proximate greenness and blood pressure were not observed among borough or city residents, although the effect size and direction were similar for townships and boroughs. Markedly higher levels of greenness and percent forest within townships compared with these other administrative community types, particularly cities, suggests the existence of a threshold effect, whereby a certain degree and/or type of greenness may be necessary to influence blood pressure in this population of individuals with T2D. Significant differences between characteristics of the study populations in each community type (e.g., socio-demographic characteristics, smoking status, BMI) raise the additional possibility of unmeasured behavioral differences that could confound the relationship between greenness and blood pressure. For example, cities may not only be less green, but city residents may also be less likely to interact with existing green spaces. Although this analysis was not designed to evaluate the specific domain by which greenness may influence blood pressure, we conjecture that townships may provide greater *restorative capacity* due to the greater percentage of forests and higher overall greenness and *reduce harm* through reduced exposure to air pollution and noise; whereas the low level of greenness in more developed community types may be insufficient to provide stress reduction or counteract such environmental stressors. The *behavioral capacity* of greenness to encourage physical activity could also be relevant but requires evaluation of greenspaces (e.g., parks) that facilitate such behaviors.

To address limitations of past studies, we evaluated interactions of greenness and percent forest, as different types of greenness have varying capacities to influence health [1,12]. Forests may be uniquely beneficial to health [17]. Studies of forest bathing suggest that experiences with the natural environment may at least confer short-term cardiovascular benefits, including lower blood pressure [15,16]. In interaction models, we found the average marginal effects for only the most highly forested areas were negatively related to SBP and DBP in townships, the greenest community type. This finding suggests a potential salutogenic role of forests. However, interaction terms in models stratified by community type did not rise to the level of statistical significance; thus, these findings should be interpreted cautiously.

Potential confounding by seasonality presented a challenge to this analysis. Blood pressure is typically highest in winter, which may be explained by levels of vitamin D3, personal environmental temperature, and seasonal variations in salt intake and physical activity [30], while greenness is at its lowest. Therefore, our observed association of higher greenness with lower SBP could be explained by the positive influence of the natural environment *or* seasonal cycles of blood pressure (or some interaction of the two). Although we were not able to fully separate these influences, several pieces of evidence suggest the greenness and blood pressure associations observed in townships were not explained by seasonal blood pressure differences alone. First, SBP and DBP were both higher on average in townships in winter months, but these relationships were not consistent for the other community types. This suggests that community characteristics, and not simply temperature and sunlight exposure, may partially explain differences in blood pressure. Second, models stratified by season potentially provide additional evidence for a threshold effect of greenness on blood pressure, again, only in townships. A model for non-winter in townships revealed a nonlinear relation between greenness and SBP, with SBP decreasing as greenness increased from a moderate to high level, as hypothesized. The low numbers of study individuals at the left end of the greenness distribution and overlapping confidence intervals (as seen in Figure 3) suggests the relation of SBP from low to moderate levels of greenness cannot be reliably interpreted.

Strengths of this study include evaluation of proximate greenness, thereby aligning the measurement of greenness with the timing of the blood pressure measurement; assessment of geographically diverse community types that ranged from rural to urban; and the development of a process to improve the accuracy of electronic health record data on current use of blood pressure medication. This study also had several limitations. First, blood pressure was not measured in controlled conditions. Clinical measurements of blood pressure are subject to “white-coat” effects, whereby patients can have inaccurate blood pressure readings due to the stress of the medical setting [33]. Unlike controlled studies with primary data collection, we used just one blood pressure measurement, following the precedent of other studies using electronic health record data [5,34]. Second, our greenness measure—NDVI buffers around individuals’ residences—did not account for quality or use of surrounding greenness, nor could we account for interactions with other greenness outside of the residential buffers. Third, we did not have information on past residential history and so were unable to determine the length of time study individual’s had lived at their current residence and experienced its specific level of greenness. This potential misclassification bias was somewhat minimized by studying the largely residentially stable population served by Geisinger [19]. Finally, our findings are subject to unmeasured confounding by air pollution and road traffic noise, which tend to be lower in greener areas; this may have resulted in overestimation of the association of NDVI and blood pressure [35].

## 5. Conclusions

The findings of this study provide further evidence for a beneficial impact of greenness on cardiovascular disease risk and highlight the potential importance of community type in this relationship. Our finding that higher greenness was associated with slightly lower blood pressure among individuals with T2D in townships, the majority of whom had a hypertension diagnosis, suggests the natural environment may have salutogenic effects even when arterial disease has been established. The lack of significant associations among borough and city residents tentatively suggest a threshold effect whereby high levels of greenness are necessary to influence blood pressure in this population. This study adds to a growing body of evidence connecting the natural environment and health, with implications for health-promoting community design.

## Figures and Tables

**Table 1:** Selected characteristics of study individuals with T2D in Pennsylvania, USA (2008–2016) at the time of blood pressure measurement during the second year following T2D diagnosis.

Characteristic	Total	Township Residents	Borough Residents	City Residents	*p*-Value
Number of individuals	9593	5853	2786	954	n/a
Number of communities	724	424	200	100	n/a
Sex, female, n (%)	4757 (49.6)	2772 (47.4)	1450 (52.1)	535 (56.1)	<0.001
Age, years, mean (SD)	56.9 (14.0)	57.7 (13.5)	56.0 (14.5)	54.6 (14.9)	<0.001
Race, white, n (%)	9346 (97.4)	5723 (97.8)	2729 (98.0)	894 (93.7)	<0.001
Non-Hispanic ethnicity, n (%)	9385 (97.8)	5750 (98.2)	2737 (98.2)	898 (94.1)	<0.001
Medical Assistance, ever, n (%)	2365 (13.2)	1090 (10.0)	868 (16.5)	407 (23.2)	<0.001
Smoking status, n (%)					<0.001
Current	1666 (17.4)	867 (14.8)	572 (20.5)	227 (23.8)	
Former	3635 (37.9)	2205 (37.7)	1068 (38.3)	362 (38.0)	
Never	4239 (44.2)	2738 (46.8)	1137 (40.8)	364 (38.2)	
Unknown	53 (0.6)	43 (0.7)	9 (0.3)	1 (0.1)	
Body mass index (kg/m^2^), ^1^ mean (SD)	35.3 (7.9)	35.0 (7.7)	35.6 (8.2)	35.9 (8.3)	<0.001
SBP (mmHg), mean (SD)	126.3 (14.1)	126.5 (14.2)	126.3 (14.0)	125.0 (14.1)	0.01
DBP (mmHg), mean (SD)	74.9 (9.4)	75.0 (9.4)	74.9 (9.3)	74.4 (9.8)	0.24
Blood pressure category, ^2^ n (%)					0.26
Normal	2414 (25.2)	1458 (24.9)	692 (24.8)	264 (27.7)	
Elevated	2260 (23.6)	1372 (23.4)	662 (23.8)	226 (23.7)	
Hypertension stage 1	3152 (32.9)	1916 (32.7)	921 (33.1)	315 (33.0)	
Hypertension stage 2	1767 (18.4)	1107 (18.9)	511 (18.3)	149 (15.6)	
Season of blood pressure measurement, n (%)					
Winter (December–February)	2250 (23.5)	1401 (23.9)	646 (23.2)	203 (21.3)	0.18
Non-winter (March–November)	7343 (76.6)	4452 (76.1)	2140 (76.8)	751 (78.7)	
Current blood pressure medication usage, n (%)	7009 (73.1)	4287 (73.2)	2060 (73.9)	662 (69.4)	0.02
Primary hypertension, n (%)	7000 (73.0)	4316 (73.7)	2027 (72.8)	657 (68.9)	0.01
Duration of hypertension to blood pressure measurement, ^3^ years, mean (SD)	7.2 (4.5)	7.4 (4.5)	7.0 (4.5)	6.6 (4.5)	<0.001
NDVI, mean (SD)	0.55 (0.17)	0.59 (0.16)	0.50 (0.15)	0.42 (0.14)	<0.001
Percent forest, mean (SD)	29.9 (25.3)	38.8 (25.5)	18.6 (18.4)	8.2 (11.9)	<0.001
CSD, SD units, mean (SD)	0.3 (2.7)	−0.6 (2.4)	1.1 (2.5)	3.3 (2.3)	<0.001

Abbreviations: CSD, community socioeconomic deprivation; NDVI, normalized difference vegetation index; SD, standard deviation. ^1^ Body mass index missing for 189 individuals. ^2^ Blood pressure categories defined as normal (<120 SBP & <80 DBP), elevated (120–129 SBP & <80 DBP), hypertension stage 1 (130–139 SBP or 80–89 DBP), hypertension stage 2 (≥140 SBP or ≥90 DBP). ^3^ Calculated among 7000 with a hypertension diagnosis.

**Table 2:** Adjusted ^1^ associations per interquartile range increase in greenness and blood pressure by administrative community type among 9593 individuals with T2D in Pennsylvania, USA (2008–2016).

	Townships Beta (95% CI)	Boroughs Beta (95% CI)	City Census Tracts Beta (95% CI)
Systolic blood pressure			
NDVI, 75th versus 25th quartile	−0.87 (−1.43, −0.30)	−0.76 (−1.64, 0.12)	−0.17 (−1.88, 1.53)
Primary hypertension diagnosis	6.21 (5.28, 7.14)	7.21 (5.87, 8.56)	4.76 (2.51, 7.01)
Current blood pressure medication	−0.76 (−1.75, 0.23)	−1.38 (−2.80, 0.04)	−0.95 (−3.57, 1.67)
Diastolic blood pressure			
NDVI, 75th versus 25th quartile	−0.41 (−0.78, −0.05)	−0.32 (−0.92, 0.28)	0.02 (−1.14, 1.17)
Primary hypertension diagnosis	3.82 (3.21, 4.43)	4.17 (3.33, 5.02)	3.14 (1.57, 4.70)
Current blood pressure medication	−1.15 (−1.79, −0.51)	−1.19 (−2.11, −0.27)	−1.18 (−2.84, 0.49)

Abbreviations: CI, confidence interval; NDVI, normalized difference vegetation index. ^1^ Models were also adjusted for age, age^2^, sex, race, ethnicity, Medical Assistance, and smoking status as described in Methods. Diastolic blood pressure models were further adjusted for age^3^.

## Data Availability

De-identified data presented in this study are available upon written request to the corresponding author and will require a data use agreement and IRB approval. The electronic health record data and linked community measures are not publicly available to protect patient confidentiality.

## Supplementary Materials

The following are available online at https://www.mdpi.com/1660-4601/18/2/614/s1, Figure S1: Process for determining current blood pressure medication status from electronic health records, Table S1: Comparison of the 9593 study individuals with 790 individuals with type 2 diabetes who were not included in the analysis after applying exclusions, Figure S2: Distribution of study individuals by proximate greenness by season (winter versus non-winter) across the three administrative community types.

## References

[1] Hartig T., Mitchell R., de Vries S., Frumkin H. (2014). Nature and health. Annu. Rev. Public Health.

[2] Markevych I., Schoierer J., Hartig T., Chudnovsky A., Hystad P., Dzhambov A.M., de Vries S., Triguero-Mas M., Brauer M., Nieuwenhuijsen M.J. (2017). Exploring pathways linking greenspace to health: Theoretical and methodological guidance. Environ. Res..

[3] Ulrich R.S., Altman I., Wohlwill J.F. (1983). Aesthetic and Affective Response to Natural Environment. Behavior and the Natural Environment.

[4] Fong K.C., Hart J.E., James P. (2018). A Review of Epidemiologic Studies on Greenness and Health: Updated Literature through 2017. Curr. Environ. Health Rep..

[5] Le-Scherban F., Ballester L., Castro J.C., Cohen S., Melly S., Moore K., Buehler J.W. (2019). Identifying neighborhood characteristics associated with diabetes and hypertension control in an urban African-American population using geo-linked electronic health records. Prev. Med. Rep..

[6] Twohig-Bennett C., Jones A. (2018). The health benefits of the great outdoors: A systematic review and meta-analysis of greenspace exposure and health outcomes. Environ. Res..

[7] Wen Y., Yan Q., Pan Y., Gu X., Liu Y. (2019). Medical empirical research on forest bathing (Shinrin-yoku): A systematic review. Environ. Health Prev. Med..

[8] Lastra G., Syed S., Kurukulasuriya L.R., Manrique C., Sowers J.R. (2014). Type 2 Diabetes Mellitus and Hypertension: An Update. Endocrinol. Metab. Clin..

[9] De Boer I.H., Bangalore S., Benetos A., Davis A.M., Michos E.D., Muntner P., Rossing P., Zoungas S., Bakris G. (2017). Diabetes and Hypertension: A Position Statement by the American Diabetes Association. Diabetes Care.

[10] Hu G., Jousilahti P., Tuomilehto J. (2007). Joint effects of history of hypertension at baseline and type 2 diabetes at baseline and during follow-up on the risk of coronary heart disease. Eur. Heart J..

[11] Shay C.M., Gooding H.S., Murillo R., Foraker R. (2015). Understanding and Improving Cardiovascular Health: An Update on the American Heart Association’s Concept of Cardiovascular Health. Prog. Cardiovasc. Dis..

[12] Wheeler B.W., Lovell R., Higgins S.L., White M.P., Alcock I., Osborne N.J., Husk K., Sabel C.E., Depledge M.H. (2015). Beyond greenspace: An ecological study of population general health and indicators of natural environment type and quality. Int. J. Health Geogr..

[13] Taylor L., Hochuli D.F. (2017). Defining greenspace: Multiple uses across multiple disciplines. Landsc. Urban Plan..

[14] White M.P., Pahl S., Ashbullby K., Herbert S., Depledge M.H. (2013). Feelings of restoration from recent nature visits. J. Environ. Psychol..

[15] Bach Pagès A., Peñuelas J., Clarà J., Llusià J., Campillo i López F., Maneja R. (2020). How Should Forests Be Characterized in Regard to Human Health? Evidence from Existing Literature. Int. J. Environ. Res. Public Health.

[16] Ideno Y., Hayashi K., Abe Y., Ueda K., Iso H., Noda M., Lee J.S., Suzuki S. (2017). Blood pressure-lowering effect of Shinrin-yoku (Forest bathing): A systematic review and meta-analysis. BMC Complement. Altern. Med..

[17] Akpinar A., Barbosa-Leiker C., Brooks K.R. (2016). Does green space matter? Exploring relationships between green space type and health indicators. Urban For. Urban Green..

[18] Hirsch A.G., Carson A.P., Lee N.L., McAlexander T., Mercado C., Siegel K., Black N.C., Elbel B., Long D.L., Lopez P. (2020). The Diabetes Location, Environmental Attributes, and Disparities Network: Protocol for Nested Case Control and Cohort Studies, Rationale, and Baseline Characteristics. JMIR Res. Protoc..

[19] Casey J.A., Savitz D.A., Rasmussen S.G., Ogburn E.L., Pollak J., Mercer D.G., Schwartz B.S. (2016). Unconventional Natural Gas Development and Birth Outcomes in Pennsylvania, USA. Epidemiology.

[20] Schwartz B.S., Pollak J.S., Poulsen M.N., Bandeen-Roche K., Moon K.A., DeWalle J., Siegel K.R., Mercado C.I., Imperatore G., Hirsch A.G. (2021). Association of community types and features in a case-control analysis of new onset type 2 diabetes across a diverse geography in Pennsylvania. BMJ Open.

[21] Casey J.A., Pollak J., Glymour M.M., Mayeda E.R., Hirsch A.G., Schwartz B.S. (2017). Measures of SES for Electronic Health Record-based Research. Am. J. Prev. Med..

[22] Schwartz B.S., Stewart W.F., Godby S., Pollak J., Dewalle J., Larson S., Mercer D.G., Glass T.A. (2011). Body mass index and the built and social environments in children and adolescents using electronic health records. Am. J. Prev. Med..

[23] Poulsen M.N., Glass T.A., Pollak J., Bandeen-Roche K., Hirsch A.G., Bailey-Davis L., Schwartz B.S. (2019). Associations of multidimensional socioeconomic and built environment factors with body mass index trajectories among youth in geographically heterogeneous communities. Prev. Med. Rep..

[24] Yeager R., Riggs D.W., DeJarnett N., Tollerud D.J., Wilson J., Conklin D.J., O’Toole T.E., McCracken J., Lorkiewicz P., Xie Z. (2018). Association Between Residential Greenness and Cardiovascular Disease Risk. J. Am. Heart Assoc..

[25] Didan K. (2015). MYD13Q1 MODIS/Aqua Vegetation Indices 16-Day L3 Global 250m SIN Grid V006 [Data set]. NASA EOSDIS Land Processes DAAC.

[26] Manson S., Schroeder J., Van Riper D., Ruggles S. (2019). IPUMS National Historical Geographic Information System.

[27] Nau C., Schwartz B.S., Bandeen-Roche K., Liu A., Pollak J., Hirsch A., Bailey-Davis L., Glass T.A. (2015). Community socioeconomic deprivation and obesity trajectories in children using electronic health records. Obesity.

[28] Yang B.Y., Markevych I., Bloom M.S., Heinrich J., Guo Y., Morawska L., Dharmage S.C., Knibbs L.D., Jalaludin B., Jalava P. (2019). Community greenness, blood pressure, and hypertension in urban dwellers: The 33 Communities Chinese Health Study. Environ. Int..

[29] Westreich D., Cole S.R. (2010). Invited commentary: Positivity in practice. Am. J. Epidemiol..

[30] Modesti P.A., Morabito M., Massetti L., Rapi S., Orlandini S., Mancia G., Gensini G.F., Parati G. (2013). Seasonal blood pressure changes: An independent relationship with temperature and daylight hours. Hypertension.

[31] Fang M. (2020). Trends in Diabetes Management Among US Adults: 1999–2016. J. Gen. Intern. Med..

[32] Hardy S.T., Loehr L.R., Butler K.R., Chakladar S., Chang P.P., Folsom A.R., Heiss G., MacLehose R.F., Matsushita K., Avery C.L. (2015). Reducing the Blood Pressure-Related Burden of Cardiovascular Disease: Impact of Achievable Improvements in Blood Pressure Prevention and Control. J. Am. Heart Assoc..

[33] Muntner P., Einhorn P.T., Cushman W.C., Whelton P.K., Bello N.A., Drawz P.E., Green B.B., Jones D.W., Juraschek S.P., Margolis K.L. (2019). Blood Pressure Assessment in Adults in Clinical Practice and Clinic-Based Research. J. Am. Coll. Cardiol..

[34] Choi Y.J., Kim S.H., Kang S.H., Kim S.Y., Kim O.J., Yoon C.H., Lee H.Y., Youn T.J., Chae I.H., Kim C.H. (2019). Short-term effects of air pollution on blood pressure. Sci. Rep..

[35] Klompmaker J.O., Janssen N.A.H., Bloemsma L.D., Gehring U., Wijga A.H., van den Brink C., Lebret E., Brunekreef B., Hoek G. (2019). Associations of Combined Exposures to Surrounding Green, Air Pollution, and Road Traffic Noise with Cardiometabolic Diseases. Environ. Health Perspect..

