# Supplementary material for: Association of Greenness with Blood Pressure among Individuals with Type 2 Diabetes across Rural to Urban Community Types in Pennsylvania, USA
Source: Int J Environ Res Public Health. 2021 Jan 13;18(2):614. doi: 10.3390/ijerph18020614 (PMC7828293; doi:10.3390/ijerph18020614)
Supplement: Supplementary file 1 [file ijerph-18-00614-s001.pdf]

Supplementary Material

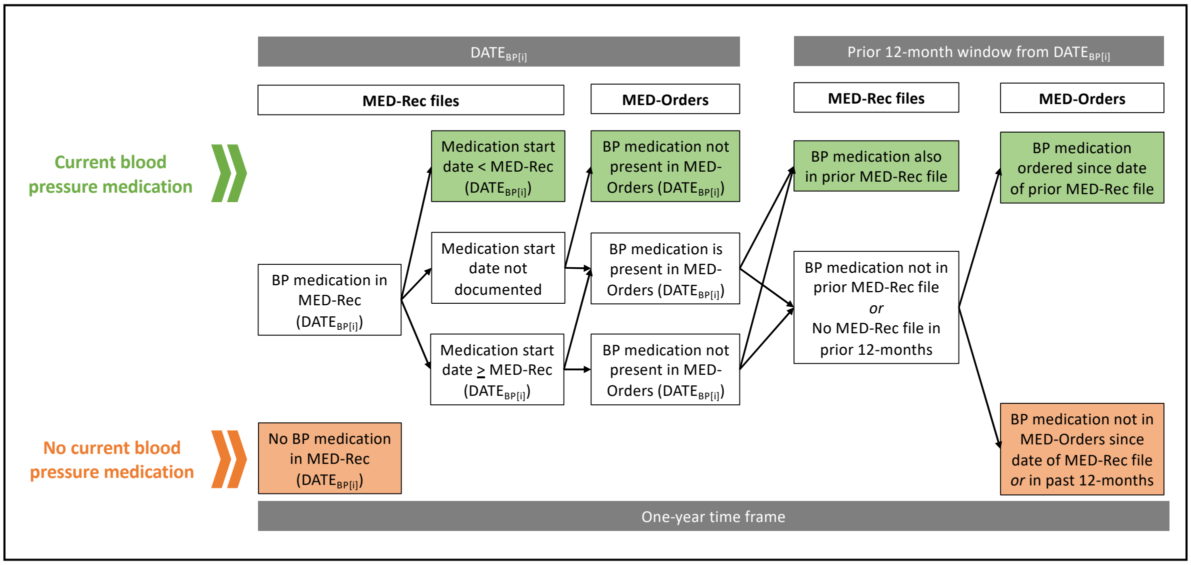

Figure S1. Process for determining current blood pressure (BP) medication status from electronic health records, given observed inconsistencies in medication reconciliation files and medication orders. Diagram presents reverse chronological order. Colored boxes depict a final determination that the study individual did (green box) or did not (orange box) currently use blood pressure medications. Medication reconciliation (MED-Rec) files (structured listing within EHR of patients' current medications as obtained during clinical encounters) from the date of the blood pressure measurement ($DATE_{BP[i]}$) were first used to determine the presence or absence of any blood pressure medication (antihypertensives including ACE inhibitors, angiotensin II receptor antagonists, beta blockers, calcium channel blockers, and diuretics). An absence of blood pressure medications from the medication reconciliation file on the date of the blood pressure measurement was considered "no current blood pressure medication use." If a medication was present, further evaluation was conducted. First, medication start dates were consulted; a start date any time prior to $DATE_{BP[i]}$ was considered current use. Next, medication orders (MED-Orders) from the date of the blood pressure measurement were consulted, as we had observed that new orders sometimes (inappropriately) appeared in the medication reconciliation file for the same day. If the medication was not present in the medication orders on the date of the blood pressure measurement, the medication was considered currently used. If present, we further evaluated whether the medication was also present in the medication reconciliation file from the most recent past encounter in the prior 12-months. If present, the medication was considered currently used. If not present, we evaluated medication orders in the prior 12-months. If the medication was present in a prior order it was considered currently used. If not, the medication was considered not currently used. This was based on the assumption that medication refills were indicated by at least one annual order, thus a lack of orders in the prior 12 months provided evidence that the medication was not currently used.

Table S1. Comparison of the 9593 study individuals with 790 individuals with type 2 diabetes who were not included in the analysis after applying exclusions[1].

Characteristic	Study Individuals	Excluded Individuals	*p*-Value [2]
Number of individuals	9593	790	n/a
Sex, female, n (%)	4757 (49.6)	371 (47.0)	0.16
Age at T2D diagnosis, years, mean (SD)	55.4 (14.0)	52.8 (20.0)	<0.001
Race, white, n (%)	9346 (97.4)	762 (96.5)	0.10
Non-Hispanic ethnicity, n (%)	9385 (97.8)	771 (97.6)	0.66

Medical Assistance, ever, n (%)	1568 (16.4)	186 (23.5)	0.001
Smoking status at T2D diagnosis, n (%)			
Current	1877 (19.6)	130 (16.5)	
Former	3290 (34.3)	270 (34.2)	0.05
Never	4285 (44.7)	372 (47.1)	
Unknown	141 (1.5)	18 (2.3)	
Body mass index at T2D diagnosis, [3] mean (SD)	36.1 (8.0)	36.3	0.56
Blood pressure medication use at T2D diagnosis, n (%)	5842 (60.9)	558 (70.6)	<0.001
Primary hypertension diagnosis at T2D diagnosis, n (%)	6578 (68.6)	623 (78.9)	<0.001

Abbreviations: SD, standard deviation; T2D, type 2 diabetes. [1] Exclusions included age <18 years (n = 96), secondary hypertension diagnosis and/or severe renal disease (n = 505), and a missing value for greenness (n = 189), as described in Methods. [2] *p*-values reflect Pearson chi-square tests for categorical variables and *t*-tests for continuous variables. [3] Body mass index missing for 622 study individuals and 36 excluded individuals with T2D.

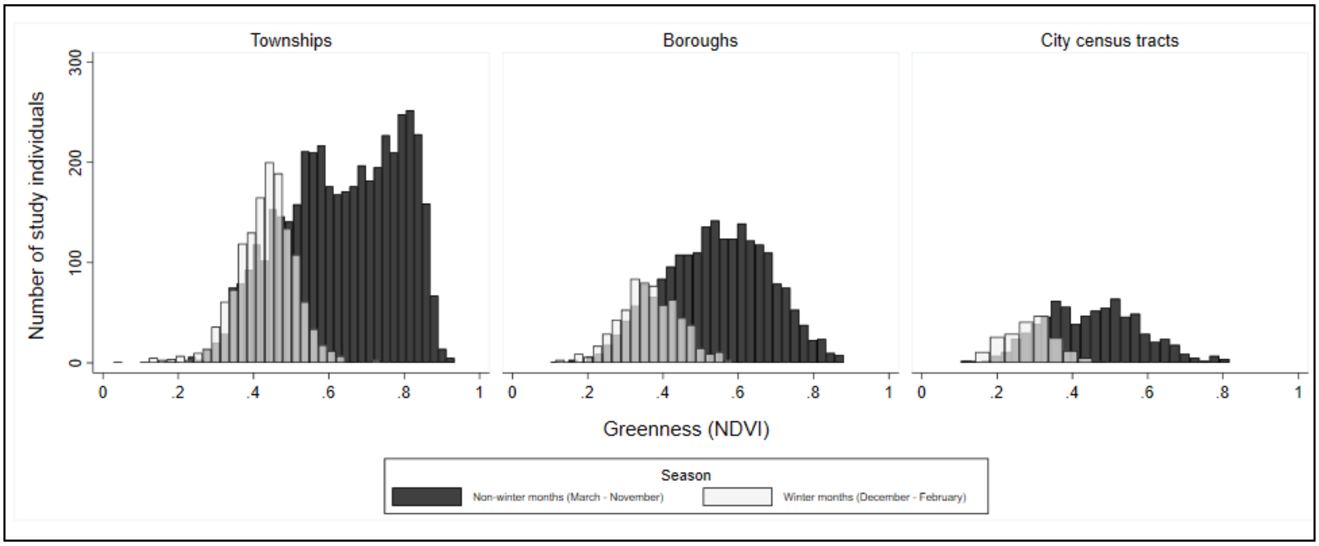

Figure S2. Distribution of study individuals by proximate greenness by season (winter versus non-winter) across the three administrative community types. Abbreviations: NDVI, normalized difference vegetation index.
